# Lacking in abundance: undernutrition in a Peri-urban fishing community in Coastal Ghana

**DOI:** 10.1186/s40795-018-0229-8

**Published:** 2018-05-02

**Authors:** Delia Akosua Bandoh, Abubakar Manu, Ernest Kenu

**Affiliations:** 1 Ghana Field Epidemiology and Laboratory Training Programme, School of Public Health, University of Ghana, Accra, Ghana; 2 School of Public Health, University of Ghana, Accra, Ghana

**Keywords:** Fishing community, Undernutrition, Diet, Stunting, Underweight, Wasting, Under-five

## Abstract

**Background:**

Malnutrition is a major problem in the world, especially in developing countries such as Ghana. Malnutrition is directly and indirectly responsible for over a million deaths in under-fives worldwide. In spite of the abundance of protein from fish and other staples in fishing communities, they are not spared of the problem of undernutrition. The study sought to assess the prevalence undernutrition among children under-five years in a fishing community in Ghana.

**Methods:**

We conducted a community-based cross-sectional study in Ekumfi Narkwa. We assessed the nutritional status of 250 children aged 6–59 months using anthropometric methods. A structured questionnaire was also used to gather socio-demographic data on the children. We generated nutritional indices with Microsoft Excel 2010 and WHO Anthro software version 3.2.2. We generate frequencies and percentages and ran a simple logistic regression to determine factors associated with malnutrition using STATA software version 13.

**Results:**

About 80%(198/250) of the children were reported to have consumed fish products more than three times in the past week whiles 85%(214/250) consumed staples more than three times in the past week. More than one-quarter (26.4%) of the children were under-nourished. The prevalence of stunting, wasting and underweight were 17.6% (44/250), 4.4% (11/250) and 12% (30/250) respectively. Age of caregiver was found to be associated with a child being underweight. The age group of the caregiver was found to be associated with undernutrition (OR:1.53; 95%CI:1.07–219).

**Conclusions:**

These findings suggest a high prevalence of under-five undernutrition in the fishing community in spite of the abundance of fish and its presence in their diet. Malnutrition interventions should therefore be extended to these communities. These interventions in fishing communities need to be tailored towards caregiver utilization of fish and other food sources available in order to improve nutrition of the children.

## Background

Malnutrition is one of the major public health concerns worldwide with developing countries facing the greatest burden [1–3]. Its consequences include retarded physical and mental growth and even death [4]. In developing countries, such as Ghana, undernutrition is the major type of malnutrition people struggle with. Each year, 35% of the deaths of children under-five is attributed to undernutrition [2, 5].

According to UNICEF, there has been a 37% drop in undernutrition, precisely low-height-for-age (stunting) worldwide since 1990 [6] . However, an evaluation of child malnutrition trends over the years points out that the decline in the rate of stunting has been slow. Therefore, millions of children still have low chances of survival if the current trends continue [7]. de Onis and his colleagues had earlier observed an uneven progress in the trend of improved malnutrition. From their observation, Africa showed very little improvement compared to Eastern and South-Eastern Asia [8]. Thus, there is the need for new ways of tackling the malnutrition in Africa to be looked into to help eradicate the problem and save millions of innocent lives.

One way of saving these lives is by improving their nutritional status through addition of nutritious foods such as fish to their diet. Fish serves as a major source of protein for many under developed countries. This is because, it is rich in proteins, essential fatty acids and micronutrients [9].

Fishing communities are characterized by the presence of fish and high consumption because of its abundance. Also, it has been recognized that household fish involvement can contribute to improving their nutritional status [10].

However, in spite of the abundance of fish in fishing communities and its presence contributing to their nutritional status, people living in fishing communities are generally thought to be at a high risk of malnutrition [11, 12]. This is because, in most of these fishing communities, fish is sold more often and not consumed by the household. Many fishing communities in developing countries of Africa and Asia are characterized by food insecurity and poverty [11].

Ghana has a wide coastline which contributes about 3.9% of the national gross domestic product (GDP) through the fishing activities and also provides part of the nation’s protein source through fish [13]. Thus, there is the need to assess the existing situation in fishing communities in Ghana to help tackle the problem of malnutrition. This would improve their health and nutritional status which is of great essence to the nation because of their significant contribution.

Assessing the nutritional status of children under five years, serves as a sensitive indicator of the overall health of the community [14, 15], thus a good tool to find out the health status of under-fives and the community as a whole.

The study sought to assess the prevalence of undernutrition among children under five years in Ekumfi Narkwa, a fishing community in the Central Region using anthropometric methods.

## Methods

### Study setting

Ekumfi Narkwa is located along the coastline of the Central region. It is one of the nine fishing communities in the district. It has a population of 4169 with 506 children under-five [16]. The community has a Community based Health Planning and Services (CHPS) compound with health workers residing in the facility. The major economic activity in the community is fishing and selling of the fresh or smoked fish. Every year, during the lean fishing season, most of the fishermen migrate to the Western region of the country to continue their fishing activities. Their wives and children go along with them to facilitate the handling and selling of the fish.

### Sample size

The sample size was determined with the following assumptions: confidence interval of 95%, a proportion of 20.2% for children under five in the Central region who are undernourished. This proportion is according to the 2014 Ghana Demographic and Health Survey [17]. The total of 250 caregiver and child pairs consented, and were recruited.

### Sampling method

We selected respondents from all the suburbs in the community namely Asemasa- Esikado, Ahenbrom, Kokodo and Adukrom. Respondents were selected proportionate to the size of the suburb. In each suburb, at least forty-five (45) respondents were selected. The proportions selected from each suburb were; Ahembrom (35%), Adukrom (25%), Esikado-Asemasa (22%) and Kokodo (18%). This was used because the community had no proper housing list [18]. Sampling of respondents from their households was done by the modified random walk method.

In the modified random walk, we identified and listed key land marks in each suburb. These included the taxi rank, clinic outreach points, schools, churches, information center, mosque, and CHPS compound. In each suburb, a listed landmark was randomly selected as the starting point for sampling of respondents. Field workers identified the house closest to the landmark and started selection of respondents from there. They continued in a clockwise manner till the sample size for the suburb was met. This procedure was repeated in each suburb till the total sample size was obtained.

In each house, we asked for caregivers with children under-five years. Every household in each sampled house with a child aged 6–59 months was given the opportunity to be part of the study. In places where a household had more than one child under five, names of both children were written on pieces of paper and the mother randomly selected one. The selected child was taken as part of the study.

The study was explained to the caregivers of these children and they were taken through the consent procedure. Caregivers who agreed signed the informed consent document before the questionnaire was administered and measurements of the child taken. In cases were the mother was less than 18 years, permission was sort form her guardian to allow her take part in the study. Again, we ensured that the guardian was present during the consent procedure and all her questions were answered. Both the mother and her guardian signed before the interviews were conducted. Participation in the study was voluntary and based on caregiver’s willingness to give full consent and the availability of the child during the study period.

### Data collection

Data was collected by interview administered questionnaires. Research assistants interviewed mothers and caregivers on their demographic characteristics, general practices, the major food groups the child had eaten in the past week and also took the child’s anthropometric measurements. Interviews and anthropometric measurements were done in the home of the caregiver.

General practices were defined as the community’s accepted way of doing things. General practices assessed were; those normally involved in childcare (who acts as caregivers), and nature of economic activities the community engages in (type of activity, nature of activity).

For the assessment of major food groups the under-five had eaten, caregivers recalled the number of times in the week the child had eaten staples (cereals, roots, tubers and plantain) and the number of times the child had taken fish products. The instrument used for food recall was adopted from the Food and Nutrition Technical Assistance (FANTA) project [19].

### Anthropometric measurements

Anthropometric measures (weight and height) were taken twice with a calibrated infantometer, ShorrBoard brand, Weigh and Measure, LLC brand and weighing scale, Seca 803 brand respectively. All measurements were recorded. All children were clothed in only underwear or light clothing during measurements. The measurements were taken using WHO standard procedure [20].

Each measurement was taken by two skilled field workers who had been trained in taking various anthropometric measurements of children under five years.

### Data analysis

Data was entered in Microsoft Excel Office 2010, cleaned and exported to STATA Software, Texas, USA, version 13 for analysis. Data collected on demographic characteristics and their general practices were presented in tables as frequencies and percentages. Means were calculated for weight and height in Microsoft Excel and were exported to WHO ANTHRO software for calculation of the nutritional indices; Height-for-age, (HA), Weight-for-age (WA) and Weight-for-Height (WH)) and their respective Z-scores. The nutritional status was assessed by the three commonly used indicators of nutritional status; height for age, weight for height and weight for age [16]. According to the WHO, child whose height for age, weight for height or weight Z-scores fell below − 2 of the mean standard deviation of the Z-score of the population was classified as stunted, wasted or underweight respectively [21]. The overall nutritional status was also determined as those whose who had Z-scores below − 2 of the population mean for any of the three nutritional indices assessed.

The anthropometric data was presented by age and sex since growth failure varies from age to age and within sexes. Age was categorized in months as: 6–11, 12–17, 18–23, 24–35, 36–47, and 48–59 respectively.

A simple logistic regression was run to assess the significant association between the nutritional status of the under-fives and the various factors assessed. Significant association was determined at 95% confidence interval and *p*-value < 0.05.

## Results

### Background characteristics of respondents

A total of 250 children between the ages of 6–59 months with their caregivers in Ekumfi Narkwa were assessed. Majority of the caregivers, 96.4% (241/250) were mothers. The average age of the caregivers interviewed was 28.7 ± 9.5 years. The youngest mother was 15 years and the oldest, 45 years. Most of the caregivers were between the ages of 20–25 years (37.6%, 94/250). The modal level of education was junior high school. The main occupation was trading of non-fish products, 55.2% (138/250) (Table 1).

**Table 1:** Socio-Demographic Characteristics of Respondents in Ekumfi Narkwa

Variable	Frequency (%)*N* = 250
Age of caregiver (years)	
Below 20	17(6.8)
20–25	94(37.6)
26–30	48(19.2)
31–35	47(18.8)
36 and above	44(17.6)
Level of education of caregiver	
No formal education	76(30.4)
Primary	67(26.8)
Junior high	97(38.8)
Senior high	8(3.2)
Tertiary	2(0.8)
Primary occupation	
Unemployed	29(11.6)
Fishmonger	35(14.0)
Farmer	15(6.0)
Trader(other than fish products)	138(55.2)
Artisan	28(11.2)
Other occupations	5(2.0)
Migration status	
Permanent resident	154(61.6)
Migrant	96(38.4)

Of the two hundred and fifty respondents, 79.2% consumed fish more than three times in the past week, whiles 85.6% consumed staples more than three times in the past week (Table 2).

**Table 2:** Food take by Under-Fives over the past week in Ekumfi Narkwa

Food group	Frequency of food groups taken	
	More than 3 times	Less than 3 times
Fish and sea foods	198(79.2)	52(20.8)
Staples	214(85.6)	36(14.4)
Vitamin A rich vegetables	84(33.6)	166(66.4)
Other vegetables	126(50.4)	124(49.6)

Half of the children surveyed were males, 50.4% (126/250). The median age group was 24–35 months (187/250). The mean age of the children was 27 months with the highest number recruited being within the ages of 12–23 months. Table 3 shows the distribution of under-fives by age and sex.

**Table 3:** Distribution of Under-Fives in Ekumfi Narkwa by Age and Sex

Age group (months)	Sex Frequency (%)		Total
	Female	Male	
6–11	23(62.2)	14(37.8)	37(14.8)
12–23	30(38.5)	48(61.5)	78(31.2)
24–35	35(48.6)	37(51.4)	72(28.8)
36–47	22(56.4)	17(43.6)	39(15.6)
48–59	14(58.3)	10(41.7)	24(9.6)
Total	124	126	250

### Nutritional status of under-fives

**Fig. 1:**
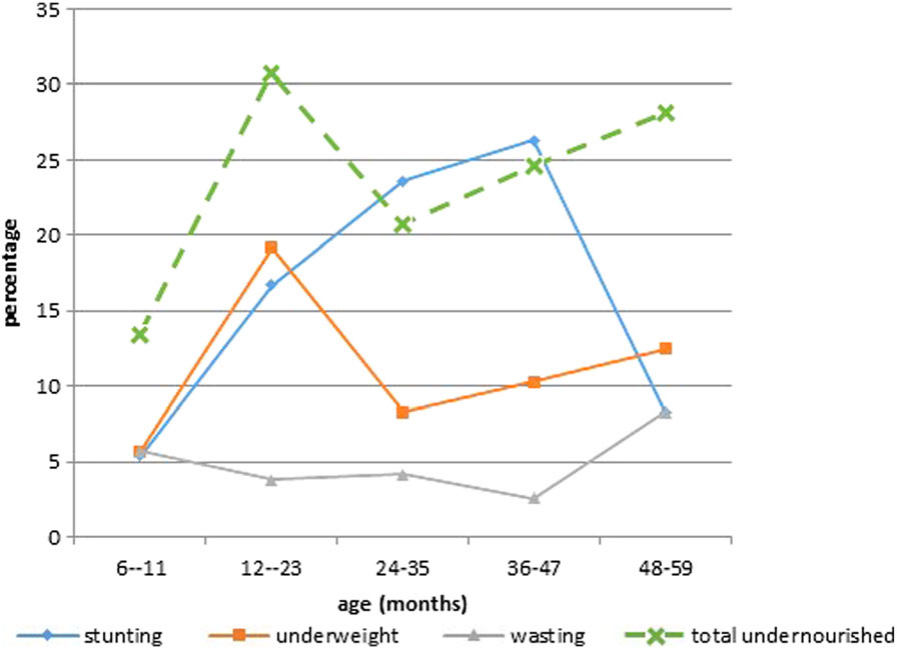
Undernutrition by age and sex

Total undernourished children were seen to follow the same growth pattern as underweight. The highest percentage was seen in children between the ages of 12-23 months. Stunting was the most common form of undernutrition whiles wasting was the least common (Fig. 1).

From Table 4, almost 18% (44/250) of the children were stunted, 12% (30/250) underweight and 4% (10/250) wasted. The mean Z-score (standard deviation) for the population from the reference group for each of the anthropometric indices were − 1.1, − 0.9 and − 0.4 for height-for-age, weight-for-age and weight-for-age respectively.

**Table 4:** Prevalence of Undernutrition among Under-fives in Ekumfi Narkwa

	Anthropometric Indices			Overall nutritional status
Category	Height-for-age (%)	Weight-for-age (%)	Weight-for-height (%)	
Low (below − 2 SD)	44 (17.6)	30 (12.0)	11 (4.4)	66 (26.4)
Normal (Above − 2 SD)	206 (82.4)	220 (88.0)	239 (95.6)	184 (73.6)
Total	250 (100)	250 (100)	250 (100)	250 (100)

From Table 5**,** Children between the age group 24-35 months had the highest level of stunting, 26.3% (17/72) whiles those within the range of 6–11 months had the lowest percentage of stunting (5%). For underweight, children within the ages of 12–23 months had the highest percentage, 19.2% (15/78) and the children between 6 and 11 months again had the lowest percentage of underweight (5.7%) that is only 2 out of the 37 children were underweight. Just 1 of the 39 children between the ages of 36–47 months was wasted. However, two of the 24 children (8.3%) between 48 and 60 months were wasted. A higher percentage of boys were stunted compared to their female counterparts (boys- 19.2% (24/125), girls- 16.1% (20/125)). However, more females were wasted than the males.

**Table 5:** Categorisation of Undernutrition among Under-fives by Age Group and Sex Distribution in Ekumfi Narkwa

N = 250 Frequency (%)						
Factors	HAZ		WAZ		WHZ	
	Stunted	Normal	Underweight	Normal	Wasted	Normal
Age (months)						
6–11	2 (5.4)	35 (94.6)	2 (5.4)	35 (94.6)	2 (5.4)	35 (94.6)
12–23	13 (16.7)	65 (83.3)	15 (19.2)	63 (80.8)	3 (3.8)	75 (96.2)
24–35	17 (23.6)	55 (76.4)	6 (8.3)	66 (91.7)	3 (4.2)	69 (85.8)
36–47	10 (25.6)	29 (74.4)	4 (10.3)	35 (89.7)	1 (2.6)	38 (97.4)
48–59	2 (8.3)	22 (91.7)	3 (12.5)	21 (87.5)	2 (8.3)	22 (91.6)
Sex						
Male	24 (19.0)	102 (80.9)	17 (13.5)	109 (87.5)	3 (2.4)	123 (97.6)
Female	20 (16.1)	104 (83.8)	13 (10.5)	111 (89.5)	8 (6.4)	116 (93.5)
Total	44 (17.6)	206 (82.4)	30 (12.0)	220 (88.0)	11 (4.4)	239 (95.6)

*HAZ* height-for-age z-score, *WAZ* weight-for-age z-score, *WHZ* weight-for-height z-score

The age of the caregiver was found to be significantly associated with being underweight (OR:1.53; 95%CI:1.07–219). The other demographic characteristics assessed were also not found to be associated with nutritional status of the under-fives (Table 6).

**Table 6:** Association between demographic characteristics and anthropometric indices assessed

Factors	Stunting		Underweight		Wasting	
	Crude OR (95% CI)	Adjusted OR (95%CI)	Crude OR (95% CI)	Adjusted OR (95% CI)	Crude OR (95% CI)	Adjusted OR (95% CI)
Caregiver age group	1.00 (0.77–1.31)	1.05 (0.83–1.38)	1.45* (1.03–2.04)	1.53* (1.07–2.19)	1.17 (0.69–1.20)	1.29 (0.76–2.20)
Caregiver’s Occupation	1.02 (0.79–1.33)	1.01 (0.78–1.32)	0.65* (0.44–0.94)	0.60 (0.962–2.42)	0.71 (0.40–1.25)	0.68 (0.37–1.23)
Caregiver’s education	1.23 (0.87–1.77)	1.23 (0.85–1.77)	1.26 (0.83–1.92)	1.53 (0.96–2.42)	1.23 (0.64–2.40)	1.39 (0.69–2.80)
Child’s sex	No observation		No observation		No observation	
Child’s age	0.85 (0.64–1.11)	1.11 (0.82–1.51)	1.03 (0.75–1.43)	1.02 (0.73–1.43)	0.94 (0.57–1.57)	0.90 (0.50–1.62)

*Significant at *p* < 0.05

## Discussion

The prevalence of undernutrition among under-fives was assessed in Ekumfi Narkwa, a fishing community along the coastline of Ghana. From the assessment, 26.4% (66/250) were under-nourished. The prevalence of stunting, underweight and wasting was 17.6%, 12% and 4.4% respectively. The prevalence for stunting and wasting obtained from this study were lower than the Ghana Demographic and Health Survey (GDHS) 2014 report. According to the GDHS, the prevalence of stunting and wasting among under-fives in Ghana are 19% and 5% respectively. However, the rate of underweight was slightly higher (12%) than the national prevalence of 11% found by the 2014 Ghana Demographic and Health Survey [17].

According to the WHO classification for severity of malnutrition, the prevalence of stunting and wasting in Ekumfi Narkwa are classified as low severity of malnutrition whiles the prevalence of underweight in the community is medium severity of malnutrition [21].

The indicator weight-for-age can be used as an overall indication of a population’s nutritional health [20]. Thus, it could be implied that about 12% of the community are not nutritionally healthy. Lack of good nutrition can lead to low productivity and inability of people to reach they maximum potential.

Stunting reflects the cumulative effects of undernutrition and infections. This indicator also reflects long term chronic restriction of a child’s growth potential. [3, 21, 22]. We observed that the prevalence of stunting was lower in the first year of life but increased through 12-35 months. This is in similar to earlier works done by Jayatissa and Badake [23, 24]. They attribute this pattern to poor weaning and complementary feeding practices leading to inadequate protein and energy intake in the child [24]. The caregiver’s lack of knowledge about proper infant and young feeding practices in spite of the abundance of the required food nutrients the child needs such as protein from fish can eventually lead to stunting of the child.

No significant differences were seen in the levels of stunting and the ages of the children (*p* < 0.05). This finding is contrary that of a study in Kenya where older children were more likely to be severely stunted as compared to younger children [25]. Since stunting is sign of prolonged lack of good nutrition, older children who have been exposed to poor nutrition for a longer period of time are more likely to be stunted than younger children.

In our study, the age of the caregiver was found to be associated with being underweight. Older mothers were 0.53 less likely to have underweight children compare to younger mothers. A study on maternal age and child nutrition also reviewed younger mothers have a higher chance of having undernourished children compared to older ones [26]. This could be due to the fact that younger mothers may lack experience and might not have been exposed to nutrition education or other nutrition interventions that may exist in the community.

The level of undernutrition in under-fives in the community, was higher than expected since there was a constant abundance of protein and energy giving foods. Again, a study in Ghana on dietary diversity in a fishing community revealed that there is an abundance of animal protein in especially from sea food and staples in their diet [27].

This finding of high level of undernutrition could be attributed to a number of reasons including the following. Most of the caregivers were traders involved in non-fish product trade. Thus, though may be an abundant commodity, there is a possibility they might not have time to obtain the food sources needed to provide meals that would meet their children’s nutrient needs. In addition to this, since a considerable number of them are migrants, they might not be able to get enough food to meet their children’s nutrient needs.

The underlying reason for the high prevalence of undernutrition could be lack knowledge on good infant and young child nutrition practices. Most caregivers might not know how the abundance of fish and other food sources available could be used to improving the nutritional status and health of the children. Education on appropriate infant feeding practices is key to solving the community’s challenge. Again, this finding could also be an indication that, the abundance of a single food commodity or food group (such as fish) required for adequate growth may not enough to improve nutritional status. According to Steyn and colleagues, a variety of food groups are required to meet the nutritional needs of the child [28]. Consumption of foods from all the essential food groups and other factors such the environment, the health of the child all contribute to the nutritional status of the child. Better child, maternal and environmental practices need to be adopted to improve young child care, growth and development and also the nutritional status of the children and the community as a whole.

This study had some limitations which include some of the following. This was a cross-sectional study thus, it was difficult to examine any potential temporal relationships or causal associations between dependent and independent variables. Furthers studies on how seasonal changes affect the nutritional status of fishing communities and the factors associated with it would be very beneficial in understanding these relationships.

## Conclusion

This study revealed that almost one-third of the children under-fives in Ekumfi Narkwa were undernourished though protein from sea foods is a stable part of their diet. This finding suggests that some fishing communities in Ghana are faced with the problem of malnutrition in spite of the abundance of protein from sea foods. This can mainly be attributed to lack of knowledge required to make good use of food sources in abundance. Malnutrition interventions mainly education on improvement of child nutrition using available food sources should be increased in these communities.

## Abbreviations

GDHS: Ghana Demographic and Health Survey
GDP: Gross domestic product
GHS: Ghana Health Service
HA: Height – for – Age
HAZ: Height – for – Age Z - Score
WA: Weight – for – Age
WAZ: Weight – for – Age Z – Score
WHO: World Health Organization
